# Detection and characterization of *Bifidobacterium crudilactis* and *B. mongoliense* able to grow during the manufacturing process of French raw milk cheeses

**DOI:** 10.1186/1471-2180-13-239

**Published:** 2013-10-29

**Authors:** Veronique Delcenserie, Bernard Taminiau, Francoise Gavini, Marie-Athenais de Schaetzen, Ilse Cleenwerck, Muriel Theves, Melanie Mahieu, Georges Daube

**Affiliations:** 1 Food Sciences Department, Faculty of Veterinary Medicine, University of Liège, Sart Tilman, B43b, B-4000 Liege, Belgium; 2 Technologie des Produits Animaux, Institut National de la recherche agronomique, 369 rue Jules Guesde, F-59651 Villeneuve d’Ascq, France; 3 BCCM/LMG Bacteria Collection, Laboratorium voor Microbiologie - Universiteit Gent, K.L. Ledeganckstraat 35, B-9000 Gent, Belgium

**Keywords:** Bifidobacteria, Crudilactis, Mongoliense, Raw milk cheeses, Sequencing, PCR-RFLP, DNA-DNA hybridization, MLSA

## Abstract

**Background:**

The study of a production chain of raw milk cheeses (St Marcellin, Vercors area, France) led to the isolation of two *Bifidobacterium* populations: *B. crudilactis* and *B. mongoliense*, that were able to grow along the production chain. The aims of this study were to further detect and characterize these bacteria along the process and evaluate the ability of some strains to survive or grow in adverse conditions.

**Results:**

Using PCR coupled with restriction fragment length polymorphism, *B. crudilactis* and *B. mongoliense* were detected in respectively 77% and 30% of St Marcellin cheeses from production chain after 21 days of ripening. They were present in more than half of all analyzed retail cheeses with counts going from 1.6 to 5 log cfu g-1 for *B. crudilactis* and 1.4 to 7 log cfu g-1 for *B. mongoliense*. *Bifidobacterium mongoliense* was sensitive to pH 2, with an observed decrease of at least 3 log for both studied strains (FR49/f/2 and FR41/2) after 1 h incubation. At pH 3, no significant decrease was observed. Good survival was observed for the same strains in presence of pancreatic juice with a decrease of less than one log. Survival of strain FR49/f/2 was better than FR41/2 with a decrease of 3 logarithms (in presence of 1% bile salts) and almost 2 logarithms (in presence of 0.5% bile salts). The genotypic analyses using total DNA-DNA hybridization, GC% content, 16S rRNA gene sequencing and multilocus sequencing analysis (MLSA) confirmed the classification of *Bifidobacterium. crudilactis* and *B. mongoliense* into two different clusters well separated from other bifidobacteria clusters.

**Conclusions:**

According to the observed characteristics such as survival in adverse conditions and their ability to grow under 12°C during the manufacturing process of the cheeses, which has never been described for bifidobacteria and which is a very interesting technological asset, these *B. crudilactis* and *B. mongoliense* strains should be further investigated for a potential use in new food or in food supplements.

## Background

The contamination of raw milk cheese production chains, using bifidobacteria as fecal indicators, was previously studied in two different cheese processes in France [1]. It has been demonstrated in that study that the species *Bifidobacterium pseudolongum* was an efficient indicator of fecal contamination from animal origin, since this species is predominant in cow feces and has never been isolated in human feces. In addition, as an indicator, *B. pseudolongum* was more sensitive than *E. coli* along the two different cheeses processes.

On the other side, bifidobacteria are also bacteria that are often desired in food products for their technological properties as thickening and stabilizing agents or for their health promoting effects. For these reasons, bifidobacteria and lactic acid bacteria are commonly added to fermented food such as fermented milk or yoghurt but also in food supplements. The positive effects encountered with bifidobacteria can be mitigated by the difficulty of some strains or species to resist or adapt to an industrial process. They prefer to grow in the absence of oxygen, which can be a challenge in industry. In addition, some strains are not able to survive the adverse conditions found in the stomach and duodenum making it difficult to exert any positive effect on the host.

The previous study of a production chain of L’Etoile du Vercors (St Marcellin cheese produced from raw cow’s milk, Vercors area, France) showed an increase of total bifidobacteria along the process and led to the isolation of two other *Bifidobacterium* populations, *B. crudilactis* and *B. mongoliense*[2,3]. These populations were able to grow along the cheese production chain. The description of the species *B. crudilactis* was based on one of those populations [4]. The other population could be phylogenetically identified as *B. mongoliense*[5] that was recently described based on two isolates obtained from a traditional fermented mare’s milk in Mongolia.

The aims of the study were (i) to further detect the two bifidobacteria populations along the L’Etoile du Vercors cheese production chain using 16S rDNA-Restriction Fragment Length Polymorphism, (ii) to evaluate the potential of *B. mongoliense* as a new food supplement or as a potential new probiotic by the study of several phenotypic characteristics such as sugar fermentation profile, growth temperature profile and aptitude to survive in adverse condition such as acidic environment or environment rich in bile salts and (iii) to perform a genotypic classification using DNA-DNA hybridization and MLSA (Multilocus Sequencing Analysis) to identify if those new populations belong to specific clusters.

## Results

### Validation of the PCR-RFLP method on pure isolates of bifidobacteria

Sixty-four strains belonging to 13 different species were already tested in a previous study using PCR-RFLP [6]. Eight additional strains of *Bifidobacterium crudilactis* and four French isolates identified as *B. mongoliense* were analyzed in this part of the work (Table 1). The RFLP patterns observed (i) with *Alu*I was named V (5-95-152-206-285-311 bp), (ii) with *Taq*I were called IX (120- 210-250-470 bp) or X (132-200-664 bp). The patterns combination V-X was attributed to *B. crudilactis* while the V-IX combination was observed for *B. mongoliense*.

**Table 1:** References and tests performed on all the strains analyzed in this study

**Strains**	**Performed tests**				
**Species/collection and reference nr**	**PCR-RFLP**	**Acidification and enzymatic tests**	**DNA-DNA relatedness**	**MLSA**	***In vitro *** **resistance to gastric acid, bile salts and pancreatic juice**
*B. mongoliense*			+		
DSM 21395^T^					
*B. mongoliense*	+	+	+	+	+
FR49/f/2					
*B. mongoliense*		+	+		
MarV3/22					
*B. mongoliense*	+	+	+		
FR39/1					
*B. mongoliense*		+	+		
MarV4/2					
*B. mongoliense*		+	+		
FR101/h/8;					
*B. mongoliense*		+	+		
MarV1/5					
*B. mongoliense*	+	+	+		
FR66/e/1					
*B. mongoliense*		+	+		
MarC1/13					
*B. mongoliense*		+	+		
MarF/3					
*B. mongoliense*		+	+		
PicD/1					
*B. mongoliense*		+	+		
FR70/g/2					
*B. mongoliense*		+	+		
FR47/2					
*B. mongoliense*	+	+	+	+	+
FR41/2					
*B. crudilactis*		+	+		
FR35/5					
*B. crudilactis*	+	+	+		
FR47/3					
*B. crudilactis*	+	+	+		
FR50/f/4					
*B. crudilactis*	+				
FR51/h/1					
*B. crudilactis*	+				
FR54/e/1					
*B. crudilactis*		+	+		
FR55/d/2					
*B. crudilactis*	+				
FR57/h/4					
*B. crudilactis*	+	+	+		
FR59/b/2					
*B. crudilactis*	+				
FR60/h/1					
*B. crudilactis*		+	+		
FR98/a/11					
*B. crudilactis*		+	+		
Brie/9					
*B. crudilactis*		+	+		
PicV/10					
*B. crudilactis*		+	+		
Reb/13					
*B. crudilactis*	+	+	+	+	
FR62/b/3					
= LMG 23609^T^					

### Detection of *Bifidobacterium crudilactis* and *B. mongoliense* in the L’Etoile du Vercors cheese process and in commercialized cheeses

- L’Etoile du Vercors cheese process (Table 2).

**Table 2:** **Number of positive samples (percentage) and mean counts (MC; log cfu ml**^**-1**^ **± standard deviation) containing *Bifidobacterium crudilactis *and *B. mongoliense *in the L’Etoile du Vercors cheese process at the different production steps**

**Species**	**RFLP types**	**Production steps (% positive samples/mean counts (log cfu ml**^**-1**^**)**				
		**Total**	**A**	**B**	**C**	**D**
		n = 176	n = 44/MC	n = 44/MC	n = 44/MC	n = 44MC
*B. crudilactis*	V-X	108 (61%)/	36 (82%)/	34 (77%)/	4 (9%)/	34 (77%)/
		2.27 ± 0.48	2.69 ± 1.57	2.63 ± 1.43	0.30 ± 1.02	3.45 ± 2.18
*B. mongoliense*	V-IX	31 (18%)/	10 (23%)/	7 (16%)/	1 (2%)/	13 (30%)/
		0.48 ± 0.7	0.61 ± 1.1	0.46 ± 1.1	0.0 ± 0.0	O.86 ± 1.7

Production steps: A, raw milk; B, after addition of rennet; C, after removal from the mold; D, ripening (D 21); n = number of cheese sample.

Out of the 176 samples from the L’Etoile du Vercors cheese process (44 samples at each step of the process: A-raw milk, B-after addition of rennet, C-after removal from the mold, D-ripening) analyzed by PCR-RFLP, 108 (61%) were V-X type positive (*B. crudilactis*) and 31 (18%) were V-IX type (*B. mongoliense*) positive. Their average counts were respectively 2.27 ± 0.48 and 0.48 ± 0.7 log cfu ml^-1^.

*Bifidobacterium crudilactis* was detected in 82% (step A) and 77% (steps B and D) of the samples, but in only 9% of the samples at step C. The species *B. mongoliense* was detected in only 18% of the total samples (31/176 samples). As observed for *B. crudilactis*, a very low percentage of samples were positive at step C (2%), while the highest percentage was found at stage D (30%).

The mean counts of *B. crudilactis* and of *B. mongoliense* were studied at each step of the production chain (Table 2). The *B. crudilactis* species presented highly significant variations (p < 0.0005). A marked decrease at step C (0.30 log cfu ml^-1^) was followed by a high increase at step D (3.45 log cfu ml^-1^). The mean counts of *B. mongoliense* were low (less than 1 log cfu ml^-1^ or g^-1^) at the different steps of the production chain (A, 0.61; B, 0.46; C, 0.00; D, 0.86).

- Commercialized raw milk cheeses (Table 3).

**Table 3:** **Number of positive samples and mean counts (log cfu ml**^**-1 **^**or g**^**-**^**1 ± standard deviation) of *Bifidobacterium crudilactis *** **and *B. mongoliense *** **in 5 trade St Marcellin cheeses and of 8 other trade raw milk cheeses**

**Species**	**Retail St Marcellin (5)**	Retail others* (8)
	**(number of positive) log cfu ml**^**-1 **^**or g**^**-1**^	**(number of positive) log cfu ml**^**-1 **^**or g**^**-1**^
*B. crudilactis*	(4/5) 3.6 ± 2.07	(3/8) 1.6 ± 2.27
*B. mongoliense*	(4/5) 4.6 ± 2.70	(5/8) 1.4 ± 1.51

*, Other retail cheese with a manufacturing process similar to the one in Vercors (Picodon, Brie and Reblochon).

Thirteen commercialized raw milk cheeses were analyzed: five trade cheeses from the plant in Vercors and eight other trade raw milk cheeses with a manufacturing process similar to the one in Vercors (Table 3).

*Bifidobacterium crudilactis* was detected in four St Marcellin cheeses from the Vercors’ plant (Day 40), with counts equal to 4 log cfu g^-1^ in two samples and 5 log cfu g^-1^ in the two others, but not in the Picodon cheese from this plant. *B. mongoliense* was detected in four cheeses out of five from this plant. This species was found in the Picodon cheese (7 log cfu g^-1^) and in three St Marcellin samples (5 log cfu^-1^ in two samples, 6 log cfu g^-1^ in the third).

In the eight other commercialized cheeses manufactured with a similar process to the one in Vercors, *B. crudilactis* was isolated from only three cheeses (mean count of 1.6 log cfu g^-1^) while *B. mongoliense* was isolated from five cheeses (mean count of 1.4 log cfu g^-1^), including one cheese made with pasteurized milk (2 log cfu g^-1^).

### Phenotypic and genotypic characterization of *Bifidobacterium crudilactis* and *B. mongoliense* strains isolated from the cheeses

The species *Bifidobacterium crudilactis* was described by [4] on the basis of 10 strains out of 141 isolates from raw milk and two raw milk cheese production chains. Of the 141 isolates, one hundred and thirty-seven were obtained from 34 raw milk cheeses produced in the same French raw milk cheese factory in the Vercors area, from April 2003 to February 2004 [4]. Another isolate was taken from a raw milk sample in another French raw milk cheese factory in the Courtenay area. The three remaining isolates came from three commercialized French raw milk cheeses (Picodon, Brie and Reblochon).

The description of *Bifidobacterium mongoliense*[5] was based on the study of two strains isolated from a traditional mare’s milk product from Mongolia. All strains analyzed in this study are presented in Table 1. Using thirteen *B. mongoliense* strains isolated from French raw milk cheeses a more complete description of the species was made. Six of these strains were isolated from six raw milk cheeses at different steps of Vercors’ plant (FR41/2 in raw milk; FR39/1 and FR47/2 after addition of rennet; FR66/e/1 after removal from the mold; FR49/f/2 during ripening at day 15; FR70/g/2 at day 21). Three strains (MarV1/5, MarV4/2 and MarV3/22) were obtained from three commercialized cheeses from the Vercors’ factory (day 40). One strain (FR101/h/8) came from a raw milk cheese sample during ripening at day 45 in another French raw milk cheese factory in the Courtenay area*,* and three others (MarC1/13, MarF/3 and PicD/1) from three commercialized French raw milk cheeses (Picodon and Saint-Marcellin). All cheeses containing *B. crudilactis* and *B. mongoliense* strains were made from raw cow milk, except for the two Picodon cheeses, which were made from raw goat milk.

The strain FR41/2 was retained as representative of the *B. mongoliense* French isolates since it was phenotypically the closest to all other French isolates.

### Phenotypic characterization

The major differential characteristics between the species *Bifidobacterium crudilactis, B. mongoliense*, and the type strain of *B. psychraerophilum* are presented in Table 4.

**Table 4:** **Phenotypic characteristics allowing differentiation between *Bifidobacterium mongoliense *** **(13 strains isolated during this study and the type strain DSM 21395**^**T**^**), *B. crudilactis *** **(10 strains, including the type strain) and *B. psychraerophilum *** **LMG 21775**^**T**^

**Characteristics**	**BM**	**BM**	**BC**	**BC**	**BP**
	**(13 strains, % positive responses)**	**DSM 21395**^**T**^[5]	**(9 strains, % positive responses; **[4]	**LMG 23609**^**T**^[4]	**LMG 21775**^**T**^[27]
**Acidification of :**					
L-arabinose	100	+	0	-	+
D-xylose	0	-	10	-	+
α-methyl-D-mannoside	0	-	0	-	+
N-acetylglu-cosamine	0	-	0	-	+
Salicin	85	+	20	-	+
Lactose	100	+	100	+	-
melezitose	0	-	10	-	+
Glycogen	92	+	10	-	-
**Enzymatic tests :**					
α-arabinosi-dase	100	NT	0	-	+
glycine arylamidase	38	NT	100	+	+
**Growth temperature range**	10°C^a^-41°C^b^	15°C-40°C	5°C-45°C	4°C-45°C	4°C-42°C
**Minimum growth pH**^**c**^	NT				4.5
**DNA G + C content (mol%)**	61.1 (6 strains) (SD = 0.67)	61.1	55.2 (9 strains) (SD = 0.83)	56.4 (4 exper.) (SD = 0.60)	59.2 (HPLC, Simpson *et al.,* 2004) 55.7 (T_m_^d^)

Legend of Table 4: **BM**: *B. mongoliense*, **BC**: *B. crudilactis*, **BP**: *B. psychraerophilum*; ^a^, growth within 14 days; ^b^, within 8 days; ^c^, within 48 h; ^d^, mean of 2 experiments performed in the laboratory; NT, not tested. Other phenotypic characteristics of the French *B. mongoliense* strains: acidification by ≥ 90% of the strains of ribose, galactose, glucose, esculin, cellobiose, maltose, melibiose and raffinose and were positive for α-galactosidase, β-galactosidase, α-glucosidase, β-glucosidase, arginine arylamidase, proline arylamidase, phenylalanine arylamidase, tyrosine arylamidase and histidine arylamidase. None (≤ 10% of the strains) ferment glycerol, erythritol, D-arabinose, L-xylose, adonitol, β-methyl-xyloside, L-sorbose, rhamnose, dulcitol, inositol, mannitol, sorbitol*,* inulin, xylitol, D-lyxose, D-tagatose, D-fucose, L-fucose, D-arabitol, L-arabitol, gluconate, 2-keto-gluconate, 5-keto-gluconate. All strains (≤ 10% of the strains) were negative for urease, indole production, nitrate reduction, arginine dihydrolase, β-galactosidase-6-phosphate, β-glucuronidase, β-N-acetylglucosaminidase, acide glutamique decarboxylase, α-fucosidase, acide pyroglutamique arylamidase, glutamyl arylamidase.

*Bifidobacterium psychraerophilum* is one of the genetically closest species also able to multiply at low temperatures. The 13 French strains identified as *B. mongoliense* were able to multiply from 10°C within 14 days to 41°C within 8 days. The FR41/2 strain, representative of the 13 strains, is also able to form colonies on TPY agar under aerobic conditions at 37°C within 3 days. The colonies reach a reduced diameter under aerobic conditions compared to the diameter, up to 1 mm, observed after 3 days under anaerobic conditions. In TPY, the minimum initial pH for growth of FR41/2 was 4.7 within 48 h (weak growth observed at pH 4.4 and no growth at pH 3.8 within 15 days).

Similar growth characteristics were observed by [5] except that no growth of *B. mongoliense* YIT 10443^T^ occurred at 10°C, nor at 40°C.

### Resistance to gastric, pancreatic juices and bile salts of *Bifidobacterium mongoliense* strains FR 49/F/2 and FR41/2

#### Gastric juice

At pH 2, a decrease of at least 3 log was observed for both studied strains after 1 h of incubation. At pH 3, a good survival of both strains was observed, even after 5 h of incubation, with no significant decrease in bifidobacteria counts (Table 5).

**Table 5:** **Resistance to biological barriers for *Bifidobacterium mongoliense *** **strains FR 49/F/2 and FR 41/2**

**Strains**	**Incubation time**	**Resistance to gastric juice**^**a**^		**Resistance to pancreatic juice**^**b**^	**Resistance to bile salts**^**c**^		
		**pH 2**	**pH 3**	**pH 8**	**1%**	**0.5%**	**0.3%**
FR49/f/2	T1 h	> 3 log	0.02 ± 0.6	0.13 ± 0.35	ND	ND	ND
	T2 h	> 3 log	0.05 ± 0.6	0.44 ± 0.05	ND	ND	ND
	T3 h	> 3 log	0.05 ± 0.6	0.46 ± 0.03	ND	ND	ND
	T4 h	> 3 log	0.16 ± 0.6	0.46 ± 0.06	ND	ND	ND
	T5 h	> 3 log	0.04 ± 0.7	0.50 ± 0.07	ND	ND	ND
	T48 h	NA	NA	NA	3.39 ± 0.34	3.07 ± 0.25	1.76 ± 0.13
FR41/2	T1 h	> 3 log	−0.04 ± 0.7	0.06 ± 0.09	ND	ND	ND
	T2 h	> 3 log	0.11 ± 0.8	0.06 ± 0.06	ND	ND	ND
	T3 h	> 3 log	0.29 ± 0.7	0.01 ± 0.15	ND	ND	ND
	T4 h	> 3 log	0.51 ± 0.6	0.02 ± 0.10	ND	ND	ND
	T5 h	> 3 log	0.61 ± 0.7	0.41 ± 0.24	ND	ND	ND
	T48 h	NA	NA		4.11 ± 0.00	3.93 ± 0.00	4.14 ± 0.00

Decrease in viable cell counts (log CFU ml^-1^) after exposure: ^a^to low pH (2 and 3) solutions during 1-5 h at 37°C, ^b^to pancreatic juice solution during 1-5 h at 37°C, ^c^to bile salts (1, 0.5 and 0.3% ) during 48 h at 37°C.

NA: not applicable.

ND: not determined.

#### Pancreatic juice

Both strains had a good survival in the presence of pancreatic juice (pH 8, Table 5). A slight decrease was observed for both of them. The strain FR49/f/2 presented a decrease of respectively 0.44, 0.46, 0.46 and 0.50 log after 2 h, 3 h, 4 h and 5 h. The strain FR41/2 presented a decrease of 0.41 log after 5 h.

#### Bile salts

In presence of a concentration of 1% bile salts, a decrease of 3.39 and 4.11 log was respectively observed for the strains FR49/f/2 and FR41/2 after 48 h. The strain FR49/f/2 was less sensitive to lower bile salts concentration with a decrease of 3.07 log and 1.76 log in presence of bile salts at a concentration of respectively 0.5% and 0.3%. The sensitivity of the strain FR41/2 to lower bile salts concentration was the same than in presence of 1% with a decrease of 3.93 log and 4.14 log with a concentration of bile salts of respectively 0.5% and 0.3% (Table 5).

#### Genotypic characteristics

All data concerning *Bifidobacterium crudilactis* (phylogenetic relationships, guanine plus cytosine content, DNA-DNA relatedness) were given by [4].

The 16S rDNA sequences of *B. mongoliense* strains FR41/2^T^, FR49/f/2, and FR101/h/8 were continuous stretches of 1452 bp respectively (Accession numbers: HV688664, HV688665, HV688666). There was 100% identity between these sequences after alignment with ClustalX [7], and a high phylogenetic relationship (99.9%) between the 16S rRNA gene sequence of FR41/2 and that of *B. mongoliense* YIT 10443^T^.

The DNA G + C content of the representative strain FR41/2 was 60.5% (average of two experiments, SD = 0.71). The average DNA G + C content of six strains in the group was 61.1% (SD = 0.67), which is also the G + C content of the *B. mongoliense* type strain (YIT 10443^T^). Those values were higher than the G + C contents of *B. psychraerophilum* LMG 21775^T^ (55.7%) and *B. crudilactis* LMG 23609^T^ (56.4%), using the *T*_m_ method. However, similar DNA G + C contents are found with the same method in the genus *Bifidobacterium,* for the species *B. cuniculi, B. gallinarum, B. pseudolongum* subsp. *pseudolongum, B. pseudolongum* subsp. *globosum* and *B. scardovii*[8].

DNA-DNA hybridizations were performed with DNAs of FR41/2, other French *B. mongoliense* isolates and the type strains of *B. mongoliense*, *B. minimum* and *B. subtile*, the closest phylogenetically related species (Table 6). The DNA-DNA relatedness (%) between *B. mongoliense* DSM 21395^T^ and FR41/2 and between FR41/2 and the other French isolates (80% to 100%) confirmed identification of these isolates as *B. mongoliense.* Strain FR41/2 showed low DNA-DNA similarity to the type strains of *B. minimum* and *B. subtile* (Table 6).

**Table 6:** **DNA-DNA relatedness (%) between DNAs of FR41/2 and other French *Bifidobacterium mongoliense *** **strains, and between FR41/2 and the type strains of *B. mongoliense, B. minimum *** **and *B subtile***

**Species/collection and reference no.**	**DNA-DNA relatedness with FR41/2**
*B. mongoliense* DSM 21395^T^	95
*B. mongoliense* FR49/f/2; MarV3/22	100
*B. mongoliense* FR39/1	95
*B. mongoliense* MarV4/2	94
*B. mongoliense* FR101/h/8; MarV1/5	93
*B. mongoliense* FR66/e/1	92
*B. mongoliense* MarC1/13	89
*B. mongoliense* MarF/3	88
*B. mongoliense* PicD/1	86
*B. mongoliense* FR70/g/2	84
*B. mongoliense* FR47/2	80
*B. minimum* DSM 20102^T^	8
*B. subtile* DSM 20096^T^	15

Legend of Table 6: DSMZ, Deutsche Sammlung von Mikroorganismen und Zellkulturen GmbH, Göttingen, Germany. The strain *Bifidobacterium mongoliense* (YIT 10443) was deposited in the DSMZ by Dr. Koichi Watanabe, Yakult Central Institute for Microbial Research, 1796, Yaho, Kunitachi, Tokyo 186–8650, Japan.

### Strains clustering using a multilocus approach

**Figure 1:**
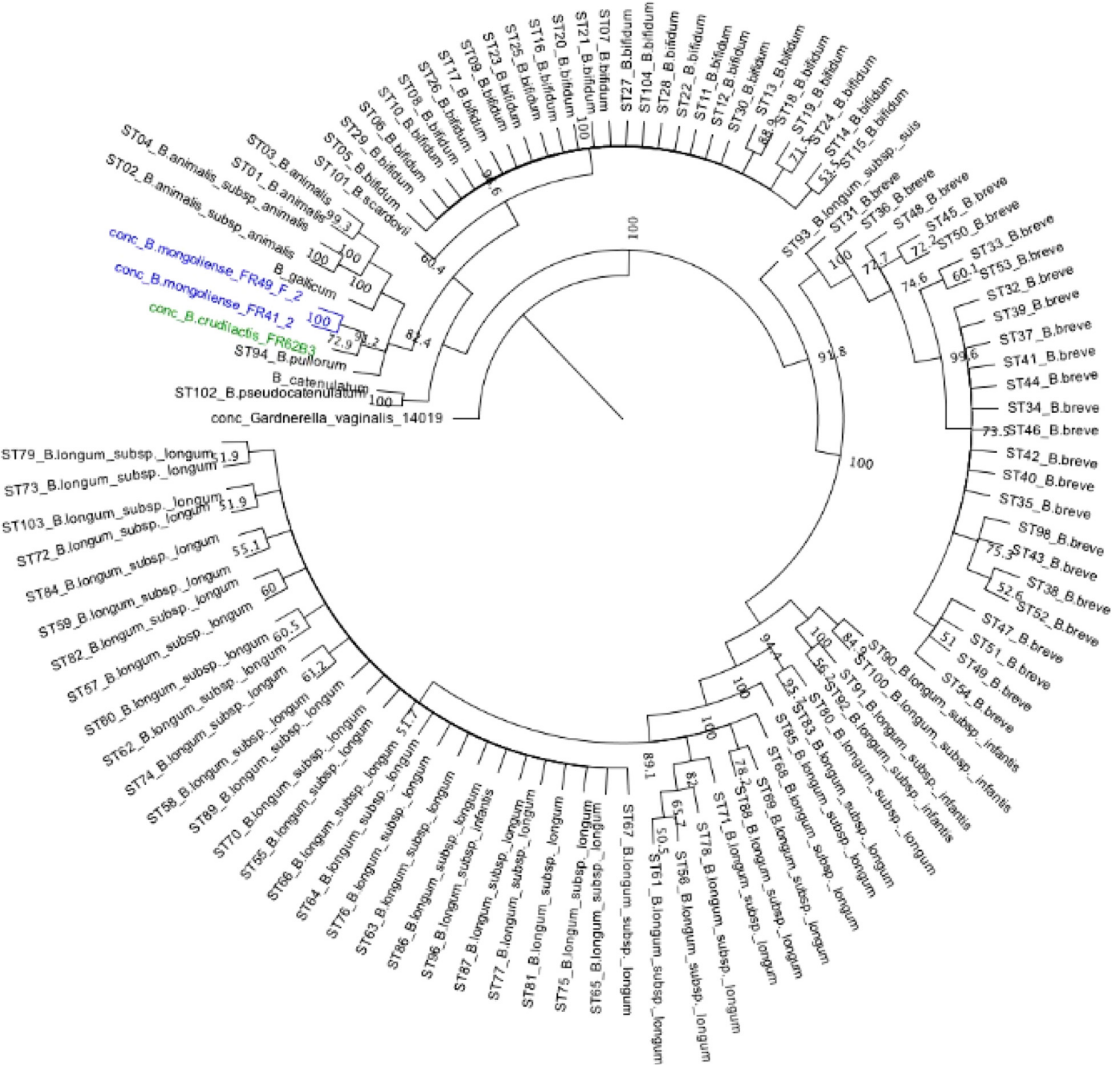
**Concatenated tree obtained by MLSA (Multilocus sequence analysis).** Phylogenetic analysis was performed based on the concatenated sequences of seven genes using the bioinformatics software “Geneious” (http://www.geneious.com).

Analysis of MLSA (Multilocus Sequencing Analysis) results of *B. mongoliense* FR41/2 and FR49/f/2 and *B. crudilactis* FR62/b/3^T^, based on concatenated sequences of the housekeeping genes *clpC*, *fusA*, *gyrB*, *ileS*, *purF*, *rplB* and *rpoB*, revealed a cluster for these strains, that was separated from other *Bifidobacterium* strains from the MLST and NCBI database (Figure 1). An allelic profile was determined for the 3 strains (Table 7), according to the method of [9] and was compared to the allelic profile of previously studied strains [9]. The same sequence type was observed for the strains FR41/2 and FR49/f/2. There were different by only one nucleic acid for the allele *purF* and *rplB*.

**Table 7:** **Sequence types of *Bifidobacterium crudilactis *** **and *B. mongoliense***

**Strains**	**Alleles**	**Template size (bp)**	**Results (Sequence type according to deletoile et al., 2010)**	**Identity %**
*B. crudilactis* FR/62/b/3	*gyrB*	600	46	79.27
	*fusA*	666	33	80.63
	*purF*	627	37	82.74
	*ileS*	489	50	82.41
	*rplB*	591	23	84.59
	*rpoB*	357	1	84.03
	*clpC*	501	33	83.42
*B. mongoliense* FR41/2	*gyrB*	600	46	80.22
	*fusA*	666	10	90.77
	*purF*	627	40	84.77
	*ileS*	489	56	88.34
	*rplB*	591	4	87.39
	*rpoB*	357	1	85.83
	*clpC*	501	9	85.24
*B. mongoliense* FR49/f/2	*gyrB*	600	46	80.22
	*fusA*	666	10	90.77
	*purF*	627	40	84.6
	*ileS*	489	56	88.34
	*rplB*	591	4	87.68
	*rpoB*	357	1	85.63
	*clpC*	501	9	85.24

## Discussion

It appears from our results that the species *Bifidobacterium crudilactis* and *B. mongoliense* are commonly present in St-Marcellin and other cheeses having a similar manufacturing process such as Picodon, Camembert, Brie and Reblochon. *B. crudilactis* and *B. mongoliense* were detected in respectively 77% and 30% of cheeses from L’Etoile du Vercors plant after 21 days of ripening. In addition, they were present in more than half of analyzed retail cheeses. Their presence and their source are not explained yet but they probably contribute to organoleptic and technologic properties of those cheeses.

During the L’Etoile du Vercors cheese process, a very low percentage of samples were positive at step C (after removal from the mold, day 2) with respectively 9% and 2% of positive samples for *Bifidobacterium crudilactis* and *B. mongoliense*. An explanation for the low percentage could be the presence of competitive flora at this stage associated with a low pH (4.35 at step C). However, during ripening (step D), the conditions seemed more favorable for those species with respectively 77% and 30% of positive samples for *B. crudilactis* and *B. mongoliense*.

The ripening temperature, 12°C, used in the Vercors factory enables these species to grow along the production chain. *B. crudilactis* was isolated at higher levels (mean count 3.45 log cfu g^-1^ at step D) than *B. mongoliense* in the Vercors’ factory. These levels were still higher in the four commercialized St Marcellin cheeses (D40, mean count 4.5 log cfu g^-1^).

*Bifidobacterium mongoliense* was detected in only 30% of the samples during ripening of the Vercors’ production chain (mean count 0.86 log cfu g^-1^ at step D) but was present at much higher levels in three of the commercialized St Marcellin cheeses (5 to 6 log cfu g^-1^) and in the Picodon cheese made with goat raw milk (7 log cfu g^-1^) from the Vercors area. These results suggest that both species, when present along the chain, continue to multiply in the cheeses after production, during storage.

*B. mongoliense* was detected in one cheese made from pasteurized milk. The most probable explanation is a cross contamination along the process or commercialization leading to the presence of this bacterial species. Interestingly, the other species, *B. crudilactis* was detected in thermized milk in our study (St Marcellin Cholet) and in thermized milk from Canada [10]. This suggests that *B. crudilactis* has the potential to resist to some heat treatment. Those properties should be better investigated, as they are important when these bacteria are incorporated in food products where heating steps are present.

The resistance to gastric and pancreatic juices, and bile salts of these bifidobacteria encourages further study for their potential to survive the transit through the human intestinal tract. The stomach normally produces around 2.5 l of HCl per day at a pH of 2.0 and a stomach pH can drop as low as 1.5 [11]. It is therefore important, when selecting probiotic strains candidates, to choose strains able to survive this barrier. Usually, acid resistance of probiotic strains is estimated for different pH values ranging from 1.5 to 5.0. Incubation time can vary from 1 to 360 minutes at 37°C, according to the fact that food usually remains in the stomach for about 90 minutes. In our study, a poor survival of strains FR49/f/2 and FR41/2 was observed in gastric juice at pH = 2, after 1 h of incubation. However, the survival of the strains was excellent at pH 3. Those results are quite encouraging knowing the limitation of the used method, namely HCl acidified media do not take into account the influence of diet or non-acidic gastric constituents on the microorganisms during their gastric transit making that test usually too severe. For example, the strain *Lactobacillus* GG cannot survive in static models, but is able to reach colon in high number *in vivo*[12,13]. The strains assessed in our study should be further tested in a dynamic *in vitro* model such as the SHIME or M-SHIME system [14,15] for confirmation of their survival through the transit.

Pancreatic secretions are amongst the most aggressive fluids in the human body. In our case, using a commercial pancreatic solution at pH 8, a good resistance of both strains was observed even after 5 h incubation.

Physiological concentrations of bile salts are between 0.3 [16] and 0.5% [17]. This is why bile solutions containing bile salts at concentrations of 0.3, 0.5 and 1% were tested in our study. Usually, bacteria are exposed to bile salts from 3 to 96 h. Bile salts secretions *in vivo* are highly variable and difficult to predict. In our study, the strain FR 49/F/2 showed a better resistance than the FR 41/2 with 0.3% bile salts. In the other conditions, a decrease of approximately 3 logarithms was observed. It is important to note that bile salts solutions and animal bile extracts are more aggressive for bacteria than human bile [16]. According to the fact that human bile is not easily available, testing of bile salts is a good compromise even if the conditions tested are probably more aggressive than in reality. The best compromise would have been to use bovine bile extract, which is less toxic than bile salts, but still more toxic than human bile.

The results obtained by MLSA are in accordance with the other genomic data presented in this study and allow a temporarily classification of *B. crudilactis* and *B. mongoliense* in two different clusters, well separated from the other bifidobacteria clusters. If, in the future, additional genome sequences are available on public database such as NCBI or on *Bifidobacterium* MLST (Multilocus sequence typing) database (Paris Institut Pasteur; [9]), more closely related *Bifidobacterium* species such as *B. bohemicum*, *B. minimum*, and *B. psychraerophilum* could be included in this analysis as well. This method was already successfully used for species identification in several genera such as *Vibrio* or *Bifidobacterium*[18-21].

However, one of the next steps will be to continue the genetic analysis of the full genome sequences to better understand the evolution of those strains and be able to explain their specific phylogenetic characteristics.

Origin of the species *B. crudilactis* and *B. mongoliense* is not known. In a previous paper [1], *B. pseudolongum* was chosen as an efficient fecal indicator since its mean counts remained stable along both studied processes. In addition, *B. pseudolongum* defined a contamination from animal origin since this species is predominant in cow dung and has never been isolated in human feces. In the case of *B. crudilactis* and *B. mongoliense*, the fecal origin is not demonstrated.

## Conclusion

The species *B. crudilactis* and *B. mongoliense* were detected in raw milk cheeses from the Vercors’ area. The origin of these bacteria is not known yet, but their presence in the cheeses probably contributes to specific organoleptic and technologic characteristics of those cheeses. This study allowed to report that these bacteria are able to grow under 12°C during the manufacturing process of the cheeses, which has never been described for bifidobacteria, and which is a very interesting technological asset. In addition, the bacteria were able to resist to low pH and aggressive fluids such as pancreatic juice and biliar salts. Because of the special characteristics of these bifidobacteria, further investigations should be performed to better characterize their technologic potential or their beneficial effect as possible new probiotics. The genotypic analyses using total DNA-DNA hybridization, GC% content, 16S rRNA gene sequencing and MLSA further confirmed the classification of *B. crudilactis* and *B. mongoliense* into two different clusters, well separated from other bifidobacteria clusters.

## Methods

### Samples from St Marcellin process (Vercors plant)

The manufacturing process was already described previously. Briefly, milk was collected on farms and stored in tanks at the plant at 4°C as already described [22]. Next, the milk was prepared for maturation by addition of cream, starter and surface flora. Temperature was increased to 22°C. Animal rennet was added (Day 0). On the next day (Day 1), the following steps were successively performed: molding, a first manual turnover, a manual salting and a second turnover. During that day, pH decreased from 6.5 to 4.3 while temperature remained stable (22°C). On the second day, cheeses were removed from the molds and a new manual or mechanical salting was performed. Ripening was then carried out for 28 days. Temperature was 12°C from Day 8. During that stage, pH slowly increased from 4.35 (at the beginning of ripening), to 4.7 (Day 15), to 5.5 (Day 21), to more than 6 (Day 28).

Forty-four raw milk cheeses at 4 different steps (176 samples) were analyzed at the following production steps: raw milk (Step A, Day 0), after addition of rennet (Step B, Day 0), after removal from the mold (Step C, Day 2) and during ripening (Step D, Day 21).

### Commercialized cheeses samples

Thirteen raw milk cheeses from retail were analyzed. Five were cheeses from L’Etoile du Vercors (St Marcellin, 4; Picodon/goat cheese, 1) collected at D + 40 to 43. Eight were additional cheeses produced with a similar process than the one of L’Etoile du Vercors’s company (St Marcellin fermier, 1, St Marcellin made from thermized milk « Cholet », 1, St Marcellin made from raw milk « Cholet », 1, Picodon de la Drôme (goat cheese) « Scoff », 1, Reblochon made from raw milk « Rochebrune », 1, Camembert made from raw milk « Lepetit », 1, Brie de Melun made from raw milk « St Faron », 1, Camembert made from pasteurized milk « Bridel », 1).

### PCR-RFLP

- Enrichment step.

The enrichment medium was carried on as described previously [22] in supplemented Brain Heart Infusion (BHI, 37 g l^-1^, Bio-Rad, Marnes-la-Coquette, France). One ml of milk or 1 g of raw milk cheese was transferred into a tube of 9 ml enrichment medium, and 1 ml of each of the ten fold appropriate sample dilutions in quarter-strength Ringer solution containing cystein chlorhydrate (0.3 g l^-1^) was also inoculated in tubes of enrichment medium in order to detect bifidobacteria in milk and raw milk cheese until the 10^-6^ dilution to get semi-quantitative counts as described previously [1]. Estimated mean counts of bifidobacteria (log cfu g^-1^) were obtained after calculating the averages of the last positives dilutions for which a value of 1 cfu ml^-1^ were attributed. Tubes were incubated at 37°C for 72 h in aerobiosis, as the bacteria were able to grow in depth because of the presence of agar in the medium.

- DNA extraction and PCR-RFLP protocol.

DNA for sequencing was extracted from culture broths obtained after the enrichment step as described previously [1] using the Wizard Genomic DNA purification kit (Promega, Madisson, WI, USA) with addition of lysozyme (10 mg/ml, Eurogentec, Seraing, Belgium).

The PCR method for the detection of the genus *Bifidobacterium* consisted of primers targeting the 16S rRNA gene followed by a digestion using 2 restriction enzymes *Alu*I and *Taq*I (Roche; Basel, Switzerland) for species detection as described previously [6]. Twenty microliters of the PCR product was restricted by 1U of enzyme in 1X buffer at 37°C for 3 h with *Alu*I and at 65°C for 3 h with *Taq*I in a total volume of 30 μl. Following the digestion, the products were analyzed by gel electrophoresis using 2.5% agarose gel. The profiles were analyzed using the Kodak 1D software (Thermolabsystems, Brussels, Belgium).

### Phenotypic characteristics

The F6PPK test was assayed on isolates as described by [23], after cell disruption by sonication (Sonopuls homogenizer HD-70, Bandelin, Berlin, Germany) for 30 sec. *Bifidobacterium crudilactis* and French *B. mongoliense* strains were discriminated in a phenotypic numerical study using the unweighted pair group method with averages (UPGMA, [24]). Phenotypic tests were 49 carbohydrate fermentations, 30 enzymatic tests performed on API 50CH and Rapid ID32A kits (BioMérieux-department API, La Balme les Grottes, France) according to the manufacturer’s instructions. The ability of the strains to grow at 46°C was performed in Tryptone Phytone Yeast (TPY) broth within 48 h [4]. The levels of similarity between strains were calculated using UPGMA (Sneath *et al.* 1973), and the Jaccard index. The numerical analysis included type strains of *Bifidobacterium* species and *Aeriscardovia aeriphila* as described previously [24].

The range of temperature for the growth of *Bifidobacterium crudilactis* and French *B. mongoliense* strains was tested in TPY broth (in water bath) at 41°C, 41.5°C, 43°C, 44°C, 45°C, 46°C and 47°C for 8 days and at 4°C, 5°C, 6°C, 7°C, 8°C, 9°C, and 10°C for 14 days. All temperatures were controlled using a temperature standard probe certified by the French Committee for Accreditation (COFRAC). Growth of FR62/b/3^T^ (*B. crudilactis*) and of FR41/2 (*B. mongoliense*) were also tested on TPY agar at 39°C under aerobic and anaerobic conditions and at 39°C in TPY broth at different pH values (3.8, 4.4 and 4.7). All strains tested in this study are presented in Table 1.

### Resistance to gastric, pancreatic juices and bile salts

#### Tolerance to acidic conditions and gastric juice

To measure the resistance to gastric juice during digestion, the method of [11] was followed. Briefly, the strains FR49/f/2 and FR41/2 were grown anaerobically in MRS for 36 h at 37°C. The pellets were collected by centrifugation at 4000 rpm and suspended in gastric juice solution (solution of 0.3% pepsin w/v (Sigma-Aldrich, Bornem, Belgium) and 0.5% NaCl w/v) adjusted to pH2 or pH 3. The solutions were incubated in anaerobic conditions during 5 h at 37°C. Plate counts were performed every hour using an automated spiral plater (Don Whitley Scientific LTD., Shipley, West Yorkshire, England). The results were compared to a blank composed of the corresponding strain in suspension in K_2_HPO_4_ buffer solution (pH 6.5) instead of gastric juice solution.

#### Tolerance to pancreatic juice

The resistance to pancreatic juice was measured as described by [11]. Briefly, the strains FR49/f/2 and FR41/2 were grown anaerobically in MRS for 36 h at 37°C. The pellets were collected by centrifugation at 4000 rpm and suspended in pancreatic juice solution (solution of 1 g pancreatin (Sigma-Aldrich, Bornem, Belgium) in 1 l of solution containing 0.5% NaCl w/v solution) adjusted to pH 8 using 5 mM NaOH [11,25]. Every hour during 5 hours, the survival of bifidobacteria was estimated using plate counts on MRS agar medium using an automated spiral plater (Don Whitley Scientific LTD., Shipley, West Yorkshire, England). The results were compared to a blank composed of the corresponding strain in suspension in K_2_HPO_4_ buffer solution (pH 6.5) instead of pancreatic juice solution.

#### Tolerance to bile salts

The resistance to bile salts was measured according to the method of [26]. Briefly, the strains FR49/f/2 and FR41/2 were inoculated in MRS supplemented with 0%, 0.3%, 0.5% and 1% of bile salts (LP0055, Oxoid). At T0 and after an incubation period of 48 h, plate counts were performed to evaluate the survival of both strains under the different bile salts concentrations. The results were compared to the strains incubated in the media containing 0% of bile salts.

### Genotypic characteristics

#### 16S rRNA gene sequencing

The 16S rRNA gene of strains FR41/2, FR49/f/2 and of FR101/h/8 was amplified and sequenced. Two 16S rDNA primers [27] were used to generate a 1452-pb 16S rDNA product. CO1 [28] was used for the 5′ end (5′ – AGTTTGATCCTGGCTCAG - 3′) and CO2 [28] for the 3′ end (5′ - TACCTTGTTACGACT - 3′). The PCRs were performed in 100-μl (each) solution containing 2 mM of each desoxynucleotide triphosphate (Eurogentec, Seraing, Belgium), 1,5 mM MgCl_2_, 5% (vol/vol) DMSO, 10 × FastStart reaction buffer, 0,4 μM (each) primer, 10 μl of DNA and 5 U of FastStart DNA polymerase (Roche Diagnostics Belgium, Vilvoorde, Belgium). The amplification was performed as previously described by Simpson *et al.* (2004). PCR amplicons were purified using an ExoSAP-IT® purification kit (USB Corporation, Ohio, USA). The recovered rDNA was sequenced using 4 forward primers: CO1 [27], Up1 (5′- AATAAGCACCGGCTAACTACG -3′), Up2 (5′- AACGGTGGATGCTGGATGTG – 3′) and Up3 (5′ – CGGTACAACGAGATGCGAC – 3′), and four reverse primers: CO2 [27], Rev1 (5′ – TCAGTCCCAATGTGGCCG -3′), Rev2 (5′ – GTATCTAATCCTGTTCGCTCCC -3′) and Rev3 (5′ – TCGAATTAATCCGCATGCTC – 3′) as described previously [4]. The primers’ Up1, Up2, Up3, Rev1, Rev2 and Rev3 binding sites were respectively at nucleotide numbers 402 to 422, 722 to 741, 1152 to 1170, 252 to 235, 702 to 681 and 872 to 853, within the 16S rRNA gene sequence of *B. crudilactis* FR62/b/3 (AY952449). The sequencing reaction was carried out after a purification step (AutoSeq G-50 (Amersham Biosciences, Ohio, USA)) using a DYEnamic™ET dye terminator cycle sequencing kit for MegaBace™ (Amersham Biosciences, Ohio, USA).

The sequences were aligned with 16S rRNA gene sequences of cultured bacteria. Sequences producing significant alignments as determined by BLAST analysis were *Bifidobacterium mongoliense*, *B. psychraerophilum*, *B. crudilactis* and *B. minimum* (comparison based on an alignment of 1452, 1428 pb, 1384 pb and 1416 pb respectively).

#### Determination of DNA G + C content

The DNA G + C content were determined by the thermal melting point method (*T*_m_ method, [29]) using a Cary 100 spectrophotometer connected to a Peltier temperature controller (Varian, France).

#### DNA-DNA hybridizations

DNA-DNA hybridizations between DNA of FR41/2 and the type strain of *B. mongoliense* DSM 21395^T^ were performed at BCCM/LMG. DNA for these hybridizations was extracted using the method described by [30], modified to a larger scale. The hybridizations were performed in the presence of 50% formamide at 47.5°C according to a modification [31,32] of the microplate method described by [33]. Reciprocal reactions (A x B and B x A) were performed, and the variation was within the limits described for this method. The DNA-DNA relatedness percentage reported for this pair of DNA is the mean of minimum 7 hybridizations.

DNA-DNA hybridizations between DNA of FR41/2 and the type strains of *B. mongoliense*, *B. subtile* and other French *B. mongoliense* isolates were performed with DNA extracted as described by [34], using achromopeptidase (5,000 U/500 mg bacteria, Sigma, France) and lysozyme (400,000 U/500 mg bacteria, MP Biomedicals, France) as lytic enzymes. This DNA was fragmented at approximately 100 MPa using an Aminco French pressure cell (Bioritech, France), and the degree of DNA-DNA relatedness was quantified from renaturation rates according to the method of [35], using a Cary 100 spectrophotometer connected to a Peltier temperature controller (Varian, France). The renaturation temperature was 25°C below the midpoint (*T*_m_) i.e. 67.3°C, according to the G + C content of FR62/b/3^T^. DNA-DNA relatedness values were calculated after 21 and 24 min of incubation. The first 3 min of renaturation, during which the temperature stabilised, were excluded from calculations. Reassociation percentages were 4 *V*_m_ – (*V*_a_ + *V*_b_) / 2 (*V*_a_ × *V*_b_) ^½^, where *V*_a_, *V*_b_ and *V*_m_ were the decreases in absorbance at 260 nm per min for organisms A and B and for an equal mixture of the two.

### Strains clustering using a multilocus approach

Three stains were analyzed using MLST (Multilocus Sequencing Typing): *B. crudilactis* type strain FR62/b/3^T^ and two representative strains of *B. mongoliense* (FR49/f/2 and FR41/2).

The strains were grown anaerobically at 37°C during 48 h in Brain Heart Infusion (BHI, Oxoid, Thermo Fisher Scientific, Aalst, Belgium) supplemented with propionic acid (5 ml/l), ferric citrate (0,5 g/l), cystein hydrochloride (0,5 g/l), yeast extract (5 g/l) at pH 5. The following seven housekeeping genes were chosen, based on their good discriminatory power for bifidobacteria, as demonstrated previously [9]: *clpC*, *fusA*, *gyrB*, *ileS*, *purF*, *rplB* and *rpoB*. The sequences of the different alleles for each gene were obtained after full genome sequencing of the three strains and were concatenated (Sequences deposited on Genbank; accession numbers: BankIt1591847 Seq1 KC527635 to BankIt1591847 Seq21 KC527655).

In order to obtain sufficient quantity of DNA for complete sequencing, the DNA extraction protocol of [36] was used. The library has been sequenced on a Roche FLX (1/8 plate) using Titanium chemistry (Roche Diagnostics, Vilvoorde, Belgium). For each gene, the alleles found in the genomes were compared to the alleles sequences obtained from the *Bifidobacterium* MLST (multilocus sequence typing) database (Paris Institut Pasteur) available for the probiotic *Bifidobacterium* species: *B. bifidum*, *B. breve, B. longum* subsp*. animalis* and *B. longum* subsp. *longum*[9] at http://www.pasteur.fr/recherche/genopole/PF8/mlst/Bifidobacterium.html.  This public database allows queries and downloads of allele sequences and allelic STs, provides several database tools and statistics and is also open to additional data on novel strains and species. The genomes of other *Bifidobacterium species* were also available on NCBI (*B. angulatum* DSM20098 (NZ_GG663536), *B. catenulatum* DSM 16992 (NZ_ABXY01000031), *B. dentium* ATCC 27678 (ABIX02000002) and *B. gallicum* DSM 20093 (NZ_ABXB03000001)). Alleles from each gene were selected using clustalW (http://www.ebi.ac.uk/) and concatenated. Next, the concatenated DNA sequences were aligned and analysed using the bioinformatics software “Geneious” (http://www.geneious.com/). Phylogenetic analysis was performed based on the concatenated sequences of the seven genes on Geneious (Figure 1).

## Competing interests

The authors declare that they have no competing interests.

## Authors’ contributions

VD carried out the PCR-RFLP, 16S rRNA gene sequencing and drafted the manuscript. In addition, she participated to the interpretation of MLST results and resistance of the strains to adverse conditions. BT participated in the design, analysis and interpretation of MLST experiments and resistance of the strains to adverse conditions. MM and MT carried out the resistance tests of strains in adverse conditions. MAdS developed and analysed the MLST experiments. IC carried out DNA-DNA hybridization. FG carried out the phenotypic characterization experiments, the DNA-DNA hybridization and participated in the design and coordination of the study. GD participated in the design and coordination of the study. All authors read and approved the final manuscript.

## References

[B1] Delcenserie V Gavini F China B Daube G *Bifidobacterium pseudolongum* are efficient indicators of animal fecal contamination in raw milk cheese industry BMC Microbiol 2011 11 178 21816092 10.1186/1471-2180-11-178 PMC3166927

[B2] Daube G Delcenserie V Gavini F Franssen C Pot B Probiotic Bifidobacterial Species USA, Europe: PCT/EP2008/058490, US 7959912,WO 2009/049932, 02-07-2008

[B3] Daube G Delcenserie V Gavini F Probiotic Bifidobacterial Species USA, Europe: PCT/EP2006/061247, US 20080274085, WO 2006/122850, 31-03-2006

[B4] Delcenserie V Gavini F Beerens H Tresse O Franssen C Daube G Description of a new species, *Bifidobacterium crudilactis* sp. nov., isolated from raw milk and raw milk cheeses Syst Appl Microbiol 2007 30 381 389 17321094 10.1016/j.syapm.2007.01.004

[B5] Watanabe K Makino H Sasamoto M Kudo Y Fujimoto J Demberel S *Bifidobacterium mongoliense* sp. nov., from airag, a traditional fermented mare’s milk product from Mongolia Int J Syst Evol Microbiol 2009 59 1535 1540 19502349 10.1099/ijs.0.006247-0

[B6] Delcenserie V Bechoux N Léonard T China B Daube G Discrimination between *Bifidobacterium* species from human and animal origin by PCR-restriction fragment length polymorphism J Food Prot 2004 67 1284 1288 15222566 10.4315/0362-028x-67.6.1284

[B7] Thompson JD Gibson TJ Plewniak F Jeanmougin F Higgins DG The CLUSTAL_X windows interface: flexible strategies for multiple sequence alignment aided by quality analysis tools Nucleic Acids Res 1997 25 4876 4882 9396791 10.1093/nar/25.24.4876 PMC147148

[B8] Biavati B Vescovo M Torriani S Bottazi V Bifidobacteria: history, ecology, physiology and applications Ann Microbiol 2000 50 117 131

[B9] Delétoile A Passet V Aires J Chambaud I Butel MJ Smokvina T Brisse S Species delineation and clonal diversity in four bifidobacterium species as revealed by multilocus sequencing Res Microbiol 2010 161 82 90 20060895 10.1016/j.resmic.2009.12.006

[B10] Rasolofo EA St-Gelais D LaPointe G Roy D Molecular analysis of bacterial population structure and dynamics during cold storage of untreated and treated milk Int J Food Microbiol 2010 138 108 118 20137820 10.1016/j.ijfoodmicro.2010.01.008

[B11] Charteris WP Kelly PM Morelli L Collins JK Development and application of an in vitro methodology to determine the transit tolerance of potentially probiotic *Lactobacillus* and *Bifidobacterium* species in the upper human gastrointestinal tract J Appl Microbiol 1998 84 759 768 9674129 10.1046/j.1365-2672.1998.00407.x

[B12] Siitonen S Vapaatalo H Salminen S Gordin A Saxelin M Wikberg R Kirkkola AL Effect of *Lactobacillus* GG yoghurt in prevention of antibiotic associated diarrhoea Ann Med 1990 22 57 59 2184848 10.3109/07853899009147243

[B13] Goldin BR Gorbach SL Saxelin M Barakat S Gualtieri L Salminen S Survival of *Lactobacillus* species (strain GG) in human gastrointestinal tract Dig Dis Sci 1992 37 121 128 1728516 10.1007/BF01308354

[B14] Molly K Vande Woestyne M Verstraete W Development of a 5-step multi-chamber reactor as a simulation of the human intestinal microbial ecosystem Appl Microbiol Biotechnol 1993 39 254 258 7763732 10.1007/BF00228615

[B15] Van den Abbeele P Roos S Eeckhaut V MacKenzie DA Derde M Verstraete W Marzorati M Possemiers S Vanhoecke B Van Immerseel F Van de Wiele T Incorporating a mucosal environment in a dynamic gut model results in a more representative colonization by lactobacilli Microb Biotechnol 2012 1 106 115 21989255 10.1111/j.1751-7915.2011.00308.x PMC3815277

[B16] Dunne C O’Mahony L Murphy L Thornton G Morrissey D O’Halloran S Feeney M Flynn S Fitzgerald G Daly C Kiely B O’Sullivan GC Shanahan F Collins JK In vitro selection criteria for probiotic bacteria of human origin: correlation with in vivo findings Am J Clin Nutr 2001 73 386S 392S 11157346 10.1093/ajcn/73.2.386s

[B17] Gómez Zavaglia A Kociubinski G Pérez P De Antoni G Isolation and characterization of bifidobacterium strains for probiotic formulation J Food Prot 1998 61 865 873 9678171 10.4315/0362-028x-61.7.865

[B18] Thompson CC Thompson FL Vicente AC Identification of *Vibrio cholerae* and *Vibrio mimicus* by multilocus sequence analysis (MLSA) Int J Syst Evol Microbiol 2008 58 617 621 18319466 10.1099/ijs.0.65461-0

[B19] Naser SM Thompson FL Hoste B Gevers D Dawyndt P Vancanneyt M Swings J Application of multilocus sequence analysis (MLSA) for rapid identification of *Enterococcus* species based on *rpoA* and *pheS* genes Microbiology 2005 151 2141 2150 16000705 10.1099/mic.0.27840-0

[B20] Bishop CJ Aanensen DM Jordan GE Kilian M Hanage WP Spratt BG Assigning strains to bacterial species via the internet BMC Biol 2009 7 3 19171050 10.1186/1741-7007-7-3 PMC2636762

[B21] Ventura M Canchaya C Del Casale A Dellaglio F Neviani E Fitzgerald GF van Sinderen D Analysis of bifidobacterial evolution using a multilocus approach Int J Syst Evol Microbiol 2006 56 2783 2792 17158978 10.1099/ijs.0.64233-0

[B22] Delcenserie V Bechoux N China B Daube G Gavini F A PCR method for detection of bifidobacteria in raw milk and raw milk cheese: comparison with culture-based methods J Microbiol Methods 2005 61 55 67 15676196 10.1016/j.mimet.2004.11.001

[B23] Scardovi V Sneath PHA, Mair NS, Sharpe ME, Holt JG Irregular nonsporing gram-negative rods: genus *Bifidobacterium*: Orla-Jensen 1924 Bergey’s manual of systematic bacteriology 1986 Baltimore: Williams and Wilkins Co 1418 1434

[B24] Sneath PHA Sokal RR Numerical taxonomy: the principles and practice of numerical classification 1973 San Francisco: W.H. Freeman

[B25] Collado MC Hernández M Sanz Y Production of bacteriocin-like inhibitory compounds by human fecal bifidobacterium strains J Food Prot 2005 68 1034 1040 15895738 10.4315/0362-028x-68.5.1034

[B26] Vinderola CG Reinheimer JA Lactic acid starter and probiotic bacteria: a comparative “in vitro” study of probiotic characteristics and biological barrier resistance Food Res Int 2003 36 895 904

[B27] Simpson PJ Ross RP Fitzgerald GF Stanton C *Bifidobacterium psychraerophilum* sp. nov. and *Aeriscardovia aeriphila* gen. nov., sp. nov., isolated from a porcine caecum Int J Syst Evol Microbiol 2004 54 401 406 15023951 10.1099/ijs.0.02667-0

[B28] Simpson PJ Stanton C Fitzgerald GF Ross RP Genomic diversity and relatedness of bifidobacteria isolated from a porcine cecum J Bacteriol 2003 185 2571 2581 12670982 10.1128/JB.185.8.2571-2581.2003 PMC152629

[B29] Marmur J Doty P Determination of the base composition of deoxyribonucleic acid from its thermal denaturation J Mol Biol 1962 5 109 118 14470099 10.1016/s0022-2836(62)80066-7

[B30] Gevers D Huys G Swings J Applicability of rep-PCR fingerprinting for differentiation of *Lactobacillus* species FEMS Microbiol Lett 2001 205 31 36 11728712 10.1111/j.1574-6968.2001.tb10921.x

[B31] Goris J Suzuki K De Vos P Nakase T Kersters K Evaluation of a microplate DNA-DNA hybridization method compared with the initial renaturation method Can J Microbiol 1998 44 1148 1153

[B32] Cleenwerck I Vandemeulebroecke K Janssens D Swings J Re-examination of the genus *Acetobacter*, with descriptions of *Acetobacter cerevisiae* sp. nov. and *Acetobacter malorum* sp. nov Int J Syst Evol Microbiol 2002 52 1551 1558 12361257 10.1099/00207713-52-5-1551

[B33] Ezaki T Hashimoto Y Yabuuchi E Fluorometric deoxyribonucleic acid-deoxyribonucleic acid hybridisation in microdilution wells as an alternative to membrane filter hybridisation in which radioisotopes are used to determine genetic relatedness among bacterial strains Int J Syst Bacteriol 1989 39 224 229

[B34] Marmur JA Procedure for isolation of deoxyribonucleic acid from microorganisms J Mol Biol 1961 3 208 218

[B35] De Ley J Cattoir H Reynaerts A The quantitative measurement of DNA hybridization from renaturation rates Eur J Biochem 1970 12 133 142 4984993 10.1111/j.1432-1033.1970.tb00830.x

[B36] Harris-Warrick RM Elkana Y Ehrlich SD Lederberg J Electrophoretic separation of bacillus subtilis genes Proc Natl Acad Sci USA 1975 72 2207 2211 806078 10.1073/pnas.72.6.2207 PMC432726

